# Identification of critical genes to predict recurrence and death in colon cancer: integrating gene expression and bioinformatics analysis

**DOI:** 10.1186/s12935-018-0640-x

**Published:** 2018-09-17

**Authors:** Xuan Long, Zhigang Deng, Guoqiang Li, Ziwei Wang

**Affiliations:** 1 Department of Gastroenterological Surgery, The First Affiliated Hospital of Chongqing Medical University, Chongqing Medical University, 1# Youyi Road, Chongqing, 400016 People’s Republic of China; 2 Department of General Surgery, Mianyang Central Hospital, Mianyang, 621000 Sichuan People’s Republic of China

**Keywords:** Biomarker, Colon cancer, VCAN

## Abstract

**Background:**

The purpose of this study was to screen the critical genes for future diagnosis and treatment of colon cancer by bioinformatics method.

**Methods:**

In this study, we used bioinformatics approaches to identify gene alteration that contribute to colon cancer progression via analysis of TCGA RNA sequencing data and other publicly GEO microarray data. The Random forest survival model was used to screen gene sets related to the prognosis in DEGs. Gene ontology and KEGG pathway enrichment analysis were performed to determine the potential function of DEGs.

**Results:**

We identified versican (VCAN), a member of the aggrecan/versican proteoglycan family, as a key regulator in human colon cancer development and progression involved in cell adhesion, proliferation, migration and angiogenesis and plays a central role in tissue morphogenesis and maintenance. Interestingly, we found that VCAN is highly over-expressed in colon cancer and increased expression of VCAN was associated with the progression of colon cancer. High VCAN levels also predict shorter overall survival of colon cancer patients. Furthermore, in vitro assays of silencing VCAN inhibit HCT116 cell proliferation and invasion.

**Conclusions:**

These data demonstrated VCAN were associated with tumorigenesis and may be as biomarker for identification of the pathological grade of colon cancer.

## Background

More than 1.2 million patients are diagnosed with colon cancer every year, and more than 600,000 die from the disease [1–4]. Incidence strongly varies globally and is closely linked to elements of a so-called western lifestyle. Incidence is higher in men than women and strongly increases with age; median age at diagnosis is about 70 years in developed countries [5–8]. Despite strong hereditary components, most cases of colon cancer are sporadic and develop slowly over several years through the adenoma-carcinoma sequence. The cornerstones of therapy are surgery, neoadjuvant radiotherapy (for patients with rectal cancer), and adjuvant chemotherapy (for patients with stage III/IV and high-risk stage II colon cancer) [9–11]. 5-year relative survival ranges from greater than 90% in patients with stage I disease to slightly greater than 10% in patients with stage IV disease. Screening has been shown to reduce colon cancer incidence and mortality, but organised screening programmes are still to be implemented in most countries [12–14].

Using high-throughput technology to analyze gene expression data can solve the current problem mentioned above. The gene expression profile of colon cancer had analyzed by microarray technique indicated many genes was a key factor affecting the disease progress [15, 16]. But few differentially expressed genes (DEGs) have been reported. Microarray technology combining with bioinformatics analysis makes it possible to comprehensively analyze the DEGs in mRNA expression level in the development and progression of colon cancer.

We identified versican (VCAN), a member of the aggrecan/versican proteoglycan family, as a key regulator in human colon cancer development and progression involved in cell adhesion, proliferation, migration and angiogenesis and plays a central role in tissue morphogenesis and maintenance. This gene is a member of the aggrecan/versican proteoglycan family. The protein encoded is a large chondroitin sulfate proteoglycan and is a major component of the extracellular matrix [17, 18]. This protein is involved in cell adhesion, proliferation, migration and angiogenesis and plays a central role in tissue morphogenesis and maintenance. Mutations in this gene are the cause of Wagner syndrome type 1 [19]. Multiple transcript variants encoding different isoforms have been found for this gene.

## Materials and methods

### Data sources analysis

Raw gene expression data and clinical profile were downloaded from The Cancer Genome Atlas Data Portal (https://gdc-portal.nci.nih.gov/search/s) and Gene Expression Omnibus dataset in National Center for Biotechnology Information (https://www.ncbi.nlm.nih.gov/geo/). “Limma” R package was used to identify DEGs between pediatric ependymoma samples and control samples. After running the “limma” package we got a matrix with 54,675 rows and 6 columns. As we knew, the logFC column gave the value of the contrast. Column *P* value was the associated *P*-value and adj.*P*-value was the *P*-value adjusted for multiple testing. In this analysis *P*-value < 0.05 and |logFC| > 2 were regarded as the cutoff criterion for EDGs.

### GO functional and pathway enrichment analysis

One of the main uses of GO is to perform enrichment analysis on gene sets. For example, given a set of genes that were up regulated under certain conditions, the enrichment analysis would find which GO terms were over-expressed or down-expressed using annotation for that gene set. KEGG pathway was a collection of manually drawn pathway maps representing our knowledge on the molecular interaction and reaction network for cellular processes, human diseases and so on. Because we had got the DEGs annotation by R package from the latest version of bioconductor (library “affy”, “limma” and “hgu133plus2.db”). So we could do GO and KEGG analysis in search tool for the retrieval of interacting genes (STRING) database version 10.0 on line.

### Immunohistochemical staining

For immunohistochemistry, slides were routinely deparaffinized and rehydrated, and then were subjected to heat-induced epitope retrieval in 0.01 mM citrate buffer (pH 6.0). Endogenous peroxidase activity was blocked for 10 min in 3% hydrogen peroxide and methanol. The slides were then incubated with rabbit Anti-VCAN polyclonal antibody (1:200; ab19345; Abcam Technology) at 4 °C overnight. Sections were then stained with DAB (Maixin. Bio, Fuzhou, China) for 5 min. Specific VCAN ISH signal was identified as brown, punctate dots and expression level was scored as Image-Pro plus 6.0 software. The intensity of staining was scored as 0 (negative), 1 (weakly positive), 2 (moderately positive), and 3 (strongly positive). According to the percentage of the positive staining area, the extent of staining was scored as 0 (0–10%), 1 (11%–30%), 2 (31%–50%), 3 (51%–70%), and 4 (71%–100%). The final staining scores (ranging from 0 to 7) of VCAN expression were divided into two groups:high expression groups (scores ≥ 3) and low expression groups (scores < 3).

### Cell proliferation and migration assay

The small interference RNA (siRNA) was designed by Sangon biotech. The siRNAs were transfected into HCT-116 cells using Lipofectamine^®^ RNAiMAX (Invitrogen) according to the manufacturer’s instructions. Colony formation assays were performed to detect HCT-116 cells cloning Capability after HCT-116 cells transfected with si-VCAN or si-NC. During migration assay, endothelial cells are placed on the upper layer of a cell permeable membrane and a solution containing the test agent is placed below the cell permeable membrane. Following an incubation period (18 h), the cells that have migrated through the membrane are stained and counted.

### Statistical analysis

For microarray analysis, differentially expressed genes were confirmed using a *P*-value threshold and FDR analysis. The threshold of truly significant miRNA was taken to be *P* value < 0.05 and FDR value < 0.05. The statistical analysis performed with the software of SPSS version 18.0 for Windows. All the data were expressed as mean ± SD. The statistical significance was evaluated by ANOVA or two-tailed t test, and the results were considered significant at a *P* value < 0.05.

## Results

### Identification of differently expressed genes (DEGs) in human colon cancer

**Fig. 1:**
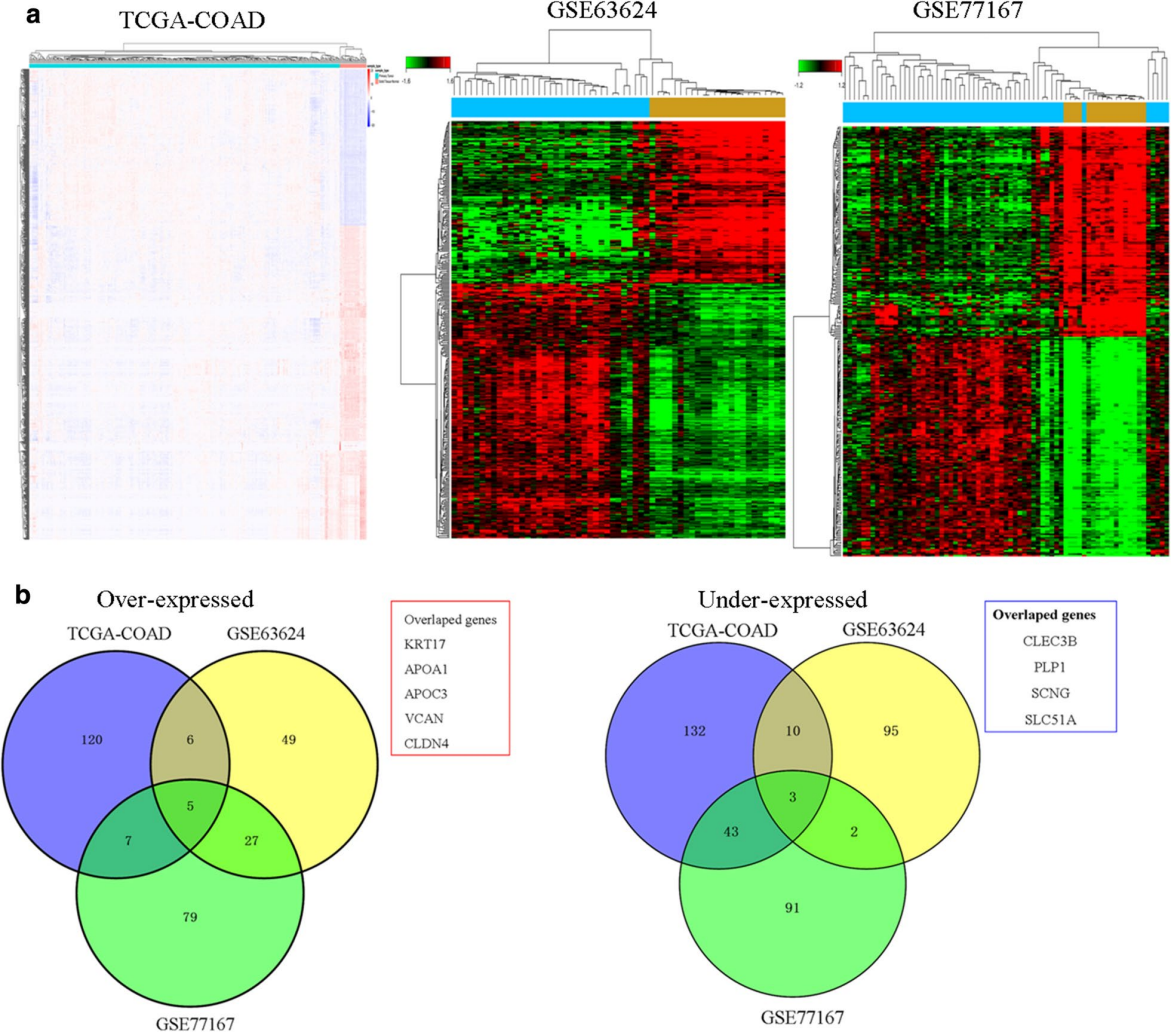
Identification of differently expressed genes (DEGs) in human colon cancer (**a**) hierarchical clustering analysis of genes that were differentially expressed (fold change > 4; *P *< 0.05) in colon cancer and normal tissues (**b**) overlap of misregulated genes in TCGA data and GEO datasets

To identify DEGs that are played key role in colon tumorigenesis, we used an integrative analysis of TCGA colon adenocarcinoma (TCGA-COAD) and RNA-seq data and colon cancer gene expression data includinging GSE63624, and GSE77167 the publicly available GEO databases. We identified 175 genes deregulated in the TCGA data, 77 in GSE63624 datasets, and 57 in GSE77167 datasets under the condition of “Q < 0.001 and fold change > 4”. Total these DEGs are shown clustered in Fig. 1a, then we founded only five genes consistently up-regulated and four down-regulated in all datasets (Fig. 1b).

### GO functional and pathway enrichment analysis

**Fig. 2:**
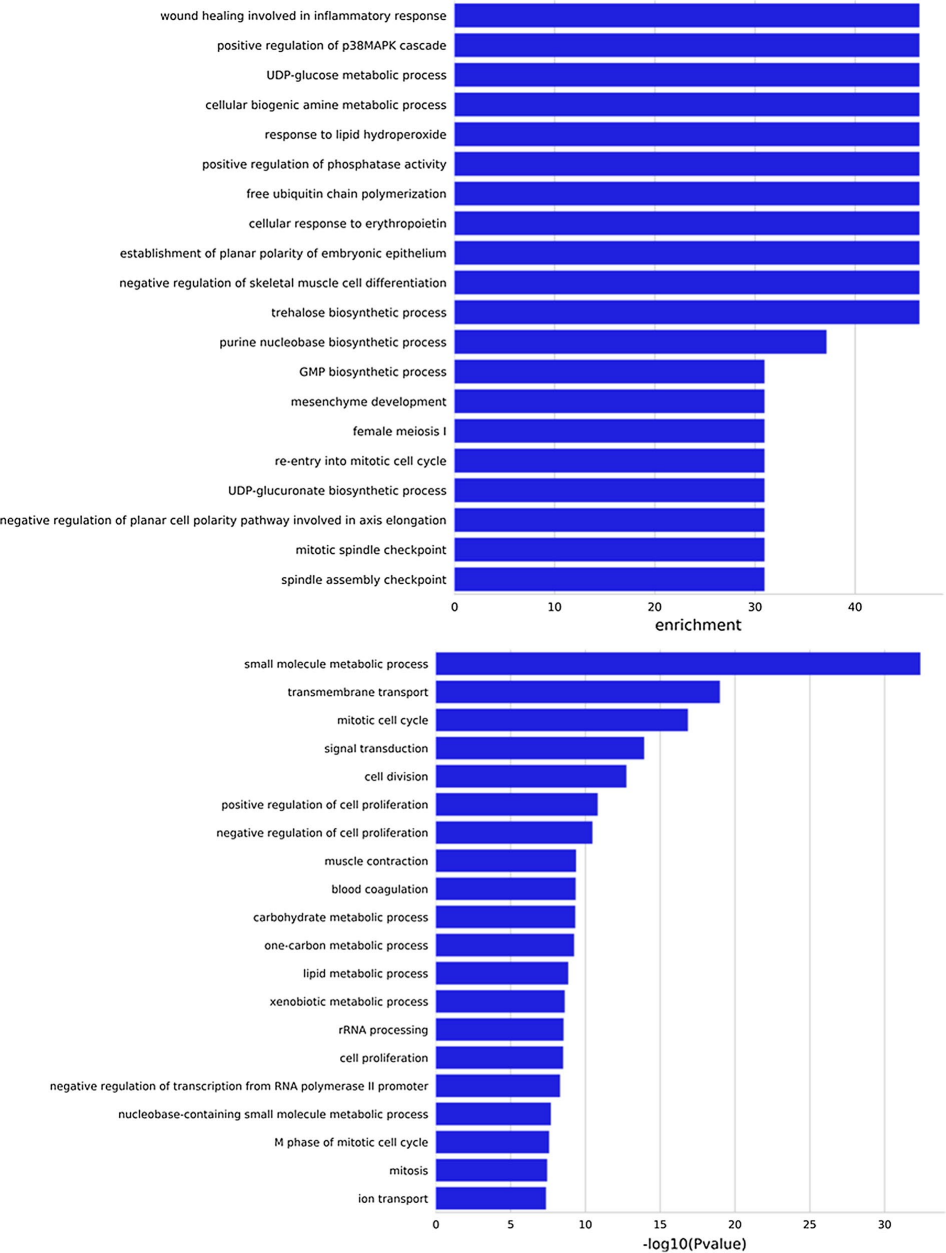
GO functional and pathway enrichment analysis of differently expressed genes (DEGs) in human colon cancer

To determine significantly DEGs in human colon cancer, gene ontology (GO) and pathway enrichment analysis were performed. We showed that the up/down-regulated DEGs were significantly enriched in wound healing involved in inflammatory response, positive regulation of phosphatase activity, cellular response to erythropoietin, negative regulation of skeletal muscle cell differentiation, mitotic cell cycle, and cell division. Important genes and pathways involved in this process are shown in Fig. 2.

### Co-expression gene-network analysis and candidate biomarker identification

**Fig. 3:**
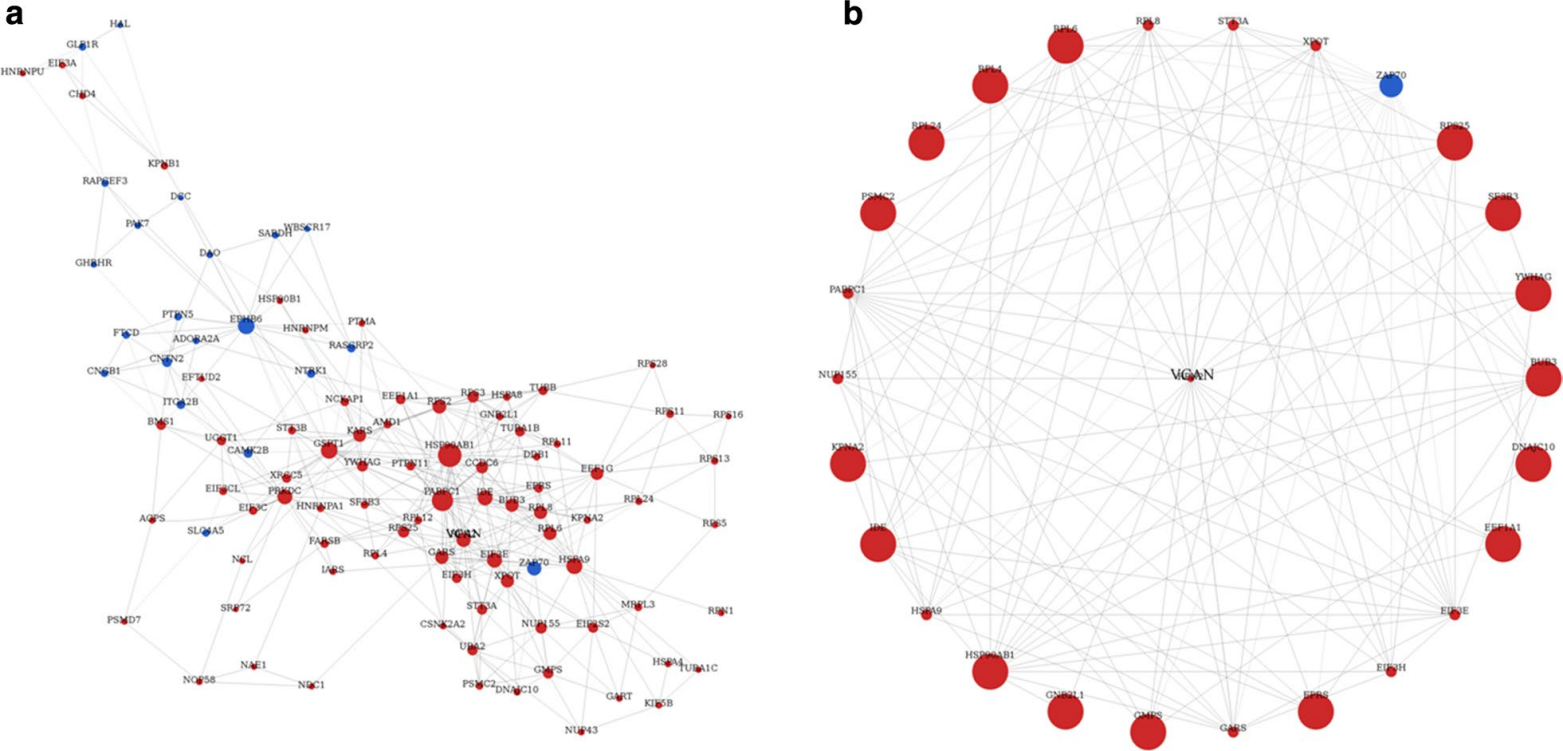
mRNA-mRNA co-expression network. **a** The differential genes were selected as candidate genes as function of IDC by constructing a gene co-expression network with k-core algorithm. **b** VCAN was the key gene in the gene network. Node size represents the degree centrality

**Fig. 4:**
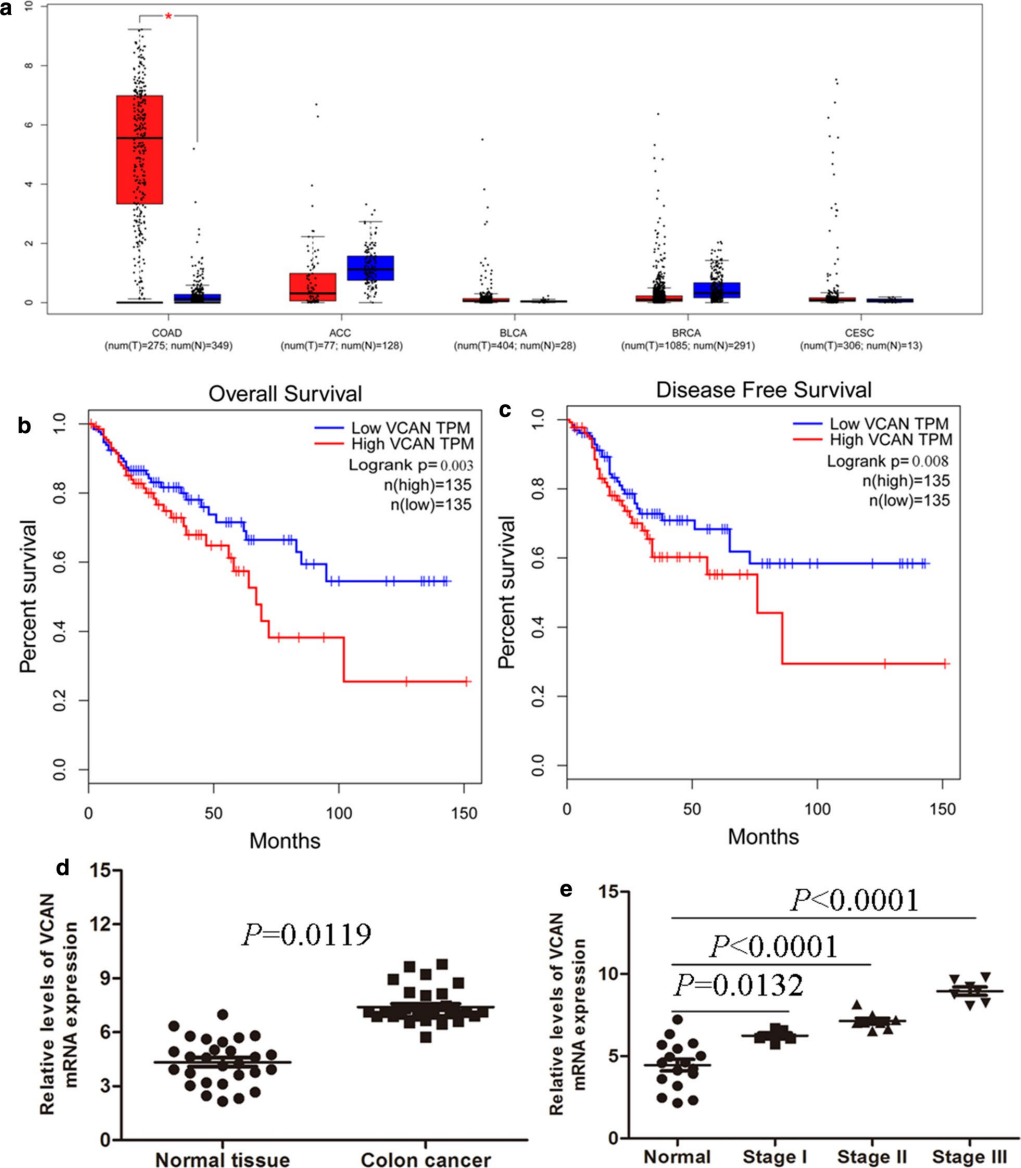
VCAN expression is elevated in primary human colon cancer. **a** Analyses of VCAN expression levels in colon adenocarcinoma (COAD), adrenocortical carcinoma (ACC), bladder urothelial carcinoma (BLCA), breast invasive carcinoma (BRCA), cervical squamous cell carcinoma and endocervical adenocarcinoma (CESC) using TCGA sequencing data. **b**, **c** Kaplan–Meier survival analysis of overall survival and disease-free survival in colon cancer patients (*P *< 0.001 for both overall survival and disease-free survival) based on VCAN expression using TCGA sequencing data. **d** VCAN expression was analyzed by qRT-PCR in colon cancer and adjacent nontumor tissues (n = 30). **e** The relationship between VCAN mRNA expression levels and clinic-pathologic parameters

To determine which gene or genes may play a pivotal role in the development of human colon cancer, we construct a gene–gene co-expression network. This co-expression network indicated HSP90AB1, VCAN, CLDN2, EPHB6, EIF3E, GSPT1, PRKDC, RPS2, GARS etc. play a key role in the progression of colon cancer (Fig. 3a). We selected VCAN over-expression genes because may be useful for early diagnosis biomarkers or therapeutic targets (Fig. 3b). We next further analysis the expression of in Colon adenocarcinoma (COAD), Adrenocortical carcinoma (ACC), Bladder Urothelial Carcinoma (BLCA), Breast invasive carcinoma (BRCA), Cervical squamous cell carcinoma and endocervical adenocarcinoma (CESC) using TCGA sequencing data and founded that is specifically upregulated in colon cancer (Fig. 4a).

### VCAN expression is elevated in primary human colon cancer

To investigate the correlation between VCAN expression and survival of colon cancer patient, we conducted a Kaplan–Meier analysis with TCGA samples dichotomized into 2 groups with expression levels less than or equal to median and levels more than median of expression. We fouded that patients in the high-risk group had significantly shorter median DFS than those in the low-risk group (Fig. 4b, c). qRT-PCR analysis were performed to detected the expression profile of VCAN in a panel of colon cancer cDNA arrays including 30 patients with colon cancer and 30 healthy controls. Result showed VCAN was significantly up-regulated at the mRNA level in colon cancer samples compared with normal colon tissues (Fig. 4d). We also founded that the expression level of VCAN mRNA was positively correlated with clinical stage (*P *< 0.05), (Fig. 4e).

### VCAN identified as a potential novel prognostic biomarker

**Fig. 5:**
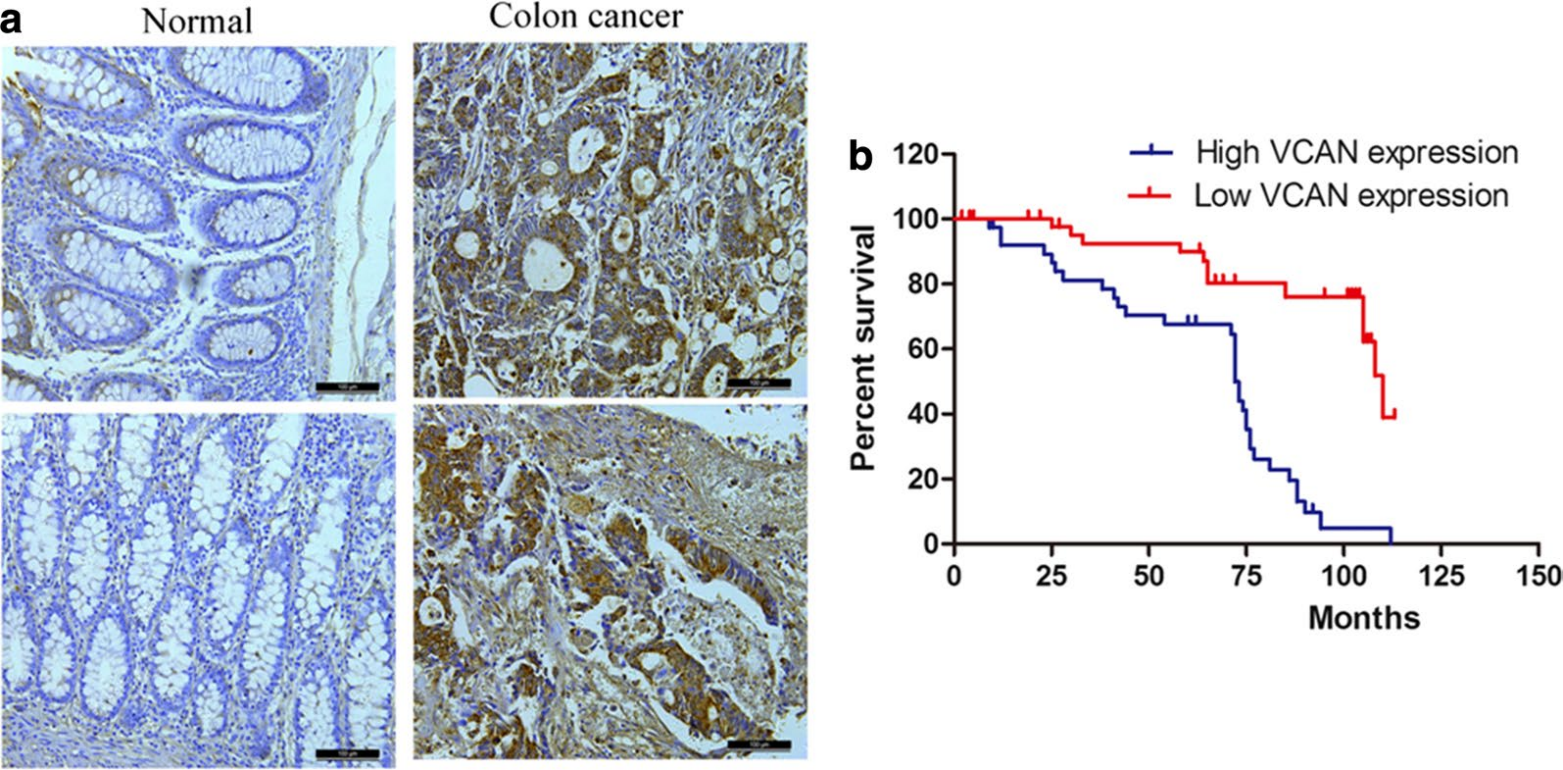
Expression of VCAN in colon cancer patient specimens. **a** Expression of VCAN in primary colon cancer and matched normal tissue (×100 or ×400). **b** Kaplan–Meier plots of VCAN expression in 37 cases of colon cancer patients. Overall survival rate was performed by log-rank test. (Immunoreactivity scores < 4 was ascribed to be low VCAN expression, immunoreactivity scores ≥ 4 was ascribed to be high VCAN expression)

Immunohistochemical staining analysis in 100 human colon cancers and matched 60 adjacent tissue microarrays showed that VCAN was significantly over-expressed in colon cancer samples compared with adjacent tissue (Fig. 5a). Kaplan–Meier survival analysis showed that the overall survival and progression-free survival rates over 3 years for the high VCAN group were lower than those in the low VCAN group (Fig. 5b). Interestingly, we founded that VCAN expression levels significantly correlated with tumor size (*P *= 0.012) and clinical stage (*P *= 0.015) in colon cancer, but not associated with other factors including pathological grading and lymph node status as shown in Table 1.

**Table 1:** Correlation of VCAN protein expression with clinicopathological data (Fisher’s exact test)

Pathological variables	Sample	VCAN IHC staining (%)		P value
		Negative	Positive	
Normal tissues	60	50 (83.3)	10 (16.7)	0.016*
Primary tumours	100	20 (20.0)	80 (80.0)	
Stage				0.015*
I + II	70	14 (20.0)	56 (80.0)	
III + IV	30	9 (30.0)	21 (70.0)	
Lymph node status				0.521
pN0	72	14 (19.4)	58 (80.6)	
pN1+	28	8 (28.5)	20 (71.5)	
Grade				0.241
1	32	6 (18.7)	26 (91.3)	
2	40	10 (25.0)	30 (75.0)	
3	28	9 (32.1)	19 (67.9)	
Tumour size				0.012
T1–3	56	12 (21.4)	44 (78.6)	
T4	44	10 (22.7)	34 (77.3)	

*P value < 0.05 was consider significant

### Silencing of VCAN inhibits HCT-116 cell colony formation and migration

**Fig. 6:**
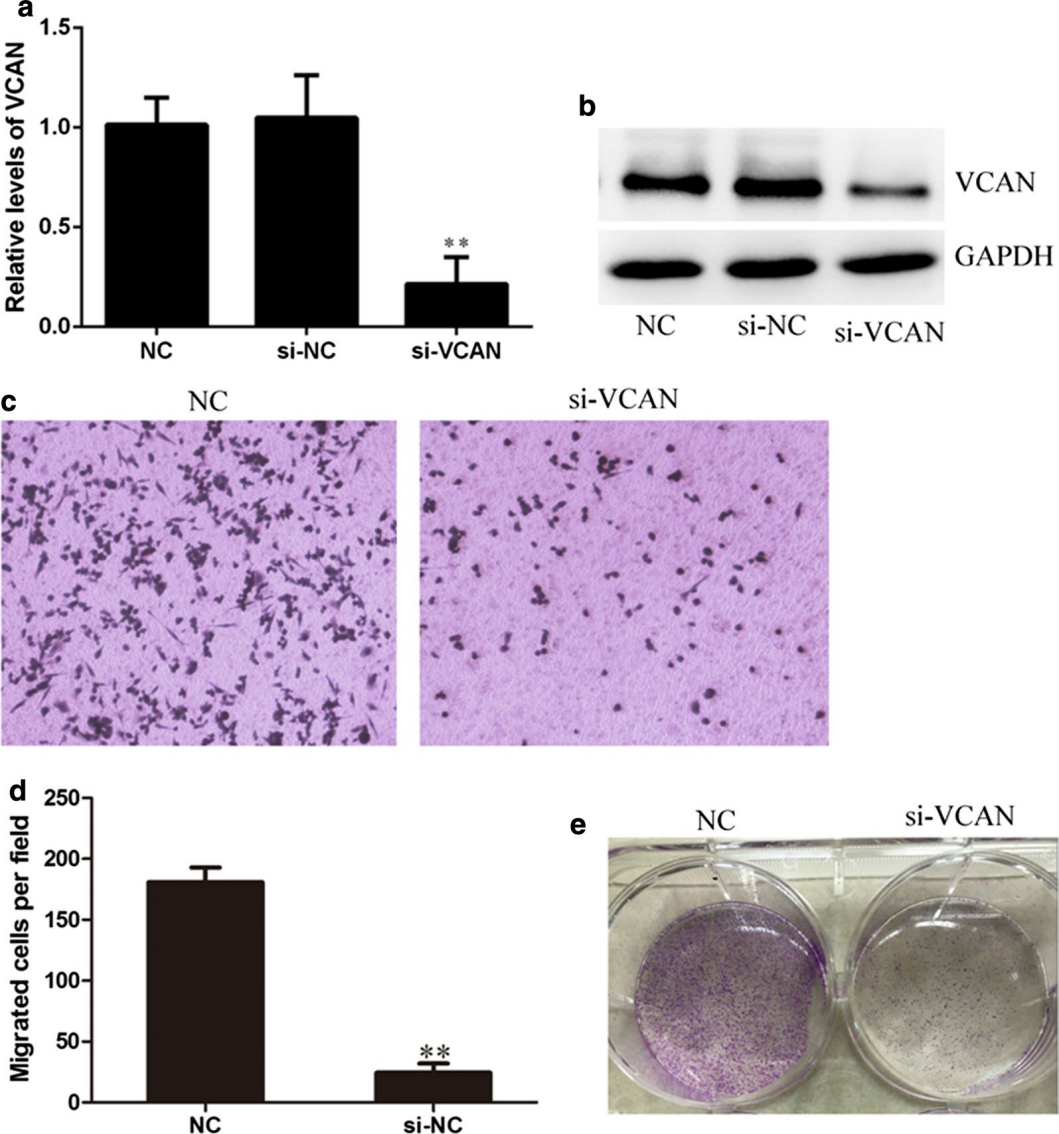
Knock-down of VCAN inhibits HCT-116 cell colony formation and migration (**a**, **b**) small interfering RNA (siRNA)-mediated knockdown ofVCAN. HCT116 cells were transfected with negative control siRNA (NC) and siRNA against VCAN (si-VCAN). After transfection, Expression of VCAN was determined by qRT-PCR and immunoblot analysis. HCT116 cells were transiently transfected with negative control siRNA and siRNA against VCAN, and then subjected to (**c**, **d**) transwell migration assay, and **e** colony formation, respectively. **p < 0.01

To determine the function of VCAN in regulating human colon cancer cell phenotype, we next performed knockdown of VCAN in HCT-116 cell line that with higher VCAN expression using small interfering RNA. Quantitative RT-PCR and Western blot analysis to quantitatively measure the effect of VCAN knockdown. Results that the VCAN expression was significantly decreased at both mRNA and protein levels in HCT116 cell lines (Fig. 6a, b). Transwell migration assays showed that knockdown of VCAN dramatically decreased cell migration (Fig. 6c, d). Furthermore, Colony formation assays showed that knockdown of VCAN inhibited cell proliferation in vitro.

## Discussion

Treatments used for colon cancer may include some combination of surgery, radiation therapy, chemotherapy and targeted therapy [20–22]. Cancers that are confined within the wall of the colon may be curable with surgery while cancer that has spread widely are usually not curable, with management being directed towards improving quality of life and symptoms [23]. Five year survival rates in the United States are around 65%. This, however, depends on how advanced the cancer is, whether or not all the cancer can be removed with surgery, and the person’s overall health. Globally, colon cancer is the third most common type of cancer making up about 10% of all cases. In 2012, there were 1.4 million new cases and 694,000 deaths from the disease [6, 24].

Previous studies demonstrate VCAN is involved in cell adhesion, proliferation, migration and angiogenesis and plays a central role in tissue morphogenesis and maintenance. Zhao et al. reported miR-135a-5p could affect the proliferation, invasion and migration of thyroid carcinoma cells by targeting VCAN [18]. Sathyan et al. reported Versican plays an important role in extracellular matrix assembly and plays a major role in the pathogenesis of IA [17]. The linkage studies also indicated VCAN as a putative candidate gene for IA in the 5q22-31 region. Chida et al. reported VCAN protein was detected exclusively in cancer stroma by immunohistochemistry, demonstrating a stepwise increase of stromal VCAN from normal tissues through stage 0 to stage IV tumors [25].

Our data demonstra that the expression level of VCAN mRNA was positively correlated with pathologic grade, clinical stage, VCAN were significantly over-expressed in metastasis samples compared with primary tumors. Immunohistochemical staining analysis in 100 human colon cancers and matched adjacent tissue microarray showed that VCAN was significantly over-expressed in colon cancer samples compared with adjacent tissue. A loss-of-function study revealed that colony formation assays showed that knockdown of VCAN inhibited cell proliferation in vitro. Transwell migration assays showed that knockdown of VCAN dramatically decreased cell migration.

## Conclusions

In conclusion, we demonstrated for the first time that VCAN is over-expressed in colorectal cancer and VCAN promotes colorectal cancer cell growth in vitro. These data suggest VCAN might serve as a potential target in the diagnosis and/or treatment in colorectal cancer.

## Abbreviations

COAD: colon adenocarcinoma
ACC: adrenocortical carcinoma
BLCA: bladder urothelial carcinoma
BRCA: breast invasive carcinoma
CESC: cervical squamous cell carcinoma and endocervical adenocarcinoma
qRT-PCR: quantitative reverse transcription polymerase chain reaction
PBS: phosphate buffered saline
TCGA: The Cancer Genome Atlas
GEO: gene expression omnibus
